# Reinforcement Learning for Radiotherapy Dose Fractioning Automation

**DOI:** 10.3390/biomedicines9020214

**Published:** 2021-02-19

**Authors:** Grégoire Moreau, Vincent François-Lavet, Paul Desbordes, Benoît Macq

**Affiliations:** Institute of Information and Communication Technologies, Electronics and Applied Mathematics, UCLouvain, 1348 Louvain-la-Neuve, Belgium; vincent.francois@uclouvain.be (V.F.-L.); paul.desbordes@uclouvain.be (P.D.); benoit.macq@uclouvain.be (B.M.)

**Keywords:** reinforcement learning, automatic treatment planning, cellular simulation

## Abstract

External beam radiotherapy cancer treatment aims to deliver dose fractions to slowly destroy a tumor while avoiding severe side effects in surrounding healthy tissues. To automate the dose fraction schedules, this paper investigates how deep reinforcement learning approaches (based on deep Q network and deep deterministic policy gradient) can learn from a model of a mixture of tumor and healthy cells. A 2D tumor growth simulation is used to simulate radiation effects on tissues and thus training an agent to automatically optimize dose fractionation. Results show that initiating treatment with large dose per fraction, and then gradually reducing it, is preferred to the standard approach of using a constant dose per fraction.

## 1. Introduction

External beam radiotherapy (EBRT) cancer treatment kills tumor cells by irradiating them [1]. The delivery of the radiation dose is fractioned so as to spare healthy cells that might be found on the beam’s trajectory. Thus, the tumor is slowly destroyed while allowing surrounding healthy tissues to recover from damage over time. The treatment schedule aims to optimize two criteria [2]:

- the tumor control probability (TCP) is the probability that the tumor is destroyed after the treatment and it should be maximized.
- the normal tissue complication probability (NTCP) is the probability that tissues and organs close to the tumor will suffer from side effects because of the treatment and it should be minimized.

To optimize these two criteria, a dosimetry step is performed prior to starting EBRT. Dose cartography is firstly optimized by the medical physician. Then, a treatment planning system splits the total dose into several fractions (with variable angles, energies, etc.). Even if daily fractions of equal dosage have been proven to produce good clinical results, recent studies claim that there could be an advantage in varying the fraction size during the treatment [3].

Deep reinforcement learning (DRL) has been able to solve a large variety of complex sequential decision-making tasks, even from high-dimensional inputs [4]. It has achieved super-human performance in many domains, such as in the game of Go [5]. In this paper, we apply these techniques to automate the fractionation task for EBRT that lead to improvements in current practice, even when using less conventional fraction schedules. Several reward functions are studied through the objectives of (i) maximizing the number of tumor cells killed and (ii) minimizing the number of healthy cells killed. Different reinforcement learning algorithms are also developed in order to study the potential of the approach.

**Figure 1:**
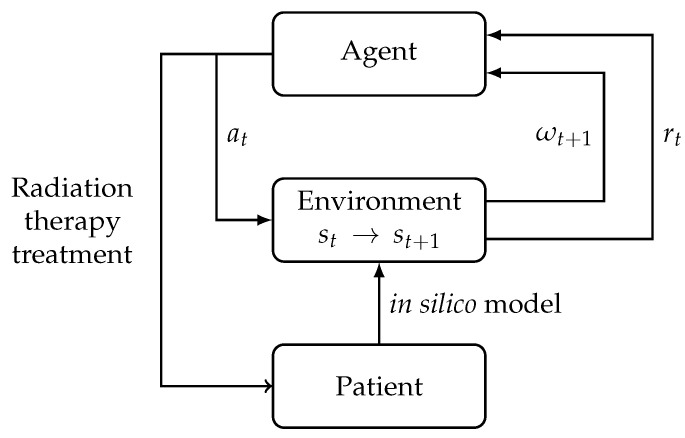
Agent–environment interaction where the environment is an in silico simulation of a tumor within its surrounding tissues. At each time step *t*, the agent takes an action $a_{t}$, which represents the dose and the environment transitions from $s_{t}$ to $s_{t+1}$, while providing a reward $r_{t}$ as well as an observation $\omega_{t+1}$.

To be optimized, a DRL algorithm needs a large amount of data, and, therefore, a model for tumor development in a healthy environment is used as a proxy. (see Figure 1). If the model is sufficiently accurate, the results can be used to modify fractionation schedules in clinical practice. Ultimately, we believe that this approach could be used in adaptive EBRT treatments for individual patients.

## 2. Related Works

### 2.1. Mathematical Modeling of Cells and Tumor Development

Cell-based computational models can be used to simulate the behaviour of human cells and their possible developments. These in silico experiments (in opposition to in vivo and in vitro) are particularly useful in cancerology, as they make it possible to study a tumor’s proliferation and reactions to treatments without resorting to expensive and time-consuming clinical experiments.

Metzcar et al. [6] published a review of the different approaches that can be used to model the development of cancer. These approaches can be split into two groups: lattice-based methods, which place cells on a grid, and off-lattice methods, which allow cells to have free positions. Several articles describing advanced simulations integrating details, such as the development of a vascular architecture around the tumor during angiogenesis [7], anaerobic respiration using H+ [8], or interactions between tumor cells and their microenvironments [9] can be found in the literature. Because our approach requires a model efficient enough to provide relatively long simulations with a reasonable computational cost, we focused on the state-of-the-art simpler models.

O’Neil et al. [10] present a simulation of tumoral development in healthy tissue. Healthy cells and cancer cells proliferate semi-independently on a 2D grid containing some glucose. Both healthy and tumor cells go through the cell cycle and can replicate if they have access to a sufficient amount of nutrients. This cycle is split into four phases: eleven hours for gap 1 (G1), eight hours for synthesis (S), four hours for gap 2 (G2), and one hour for mitosis (M). Each hour represents a time step for the simulation (discrete time). Healthy cells can also enter quiescence (G0), if the conditions in the environment are not favorable. G0 cells consume less nutrients and do not replicate. Initially, healthy cells are placed randomly on the grid. Step after step, the cancer cells spread through the entire grid to form a tumor.

Radiation therapy is also implemented in the model to enable us to prevent the spread of tumor cells as well as simulate side effects on healthy cells. It is represented by applying a survival probability of 37% to each cell along a certain radius of a center of radiation specified by the user. Cells that are not directly killed by the radiation keep count of the doses that they have received and have to repair the radiation damage over time. The radiation dose is then repeated every 24 h of simulation. To keep the model as simple as possible, the EBRT is quite naive. Furthermore, the authors did not take dioxygen into account, despite its major role in radiation therapy (such as the radio-sensitivity of cells).

Later, Jalalimanesh et al. [11] extended this simulation. First, they replaced glucose by dioxygen as the main nutrient on the grid. Second, they used a modified linear-quadratic (LQ) model to represent the effects of different intensities of radiation on cells. The LQ model [12] aims to describe the surviving fraction (SF) of a group of cells subjected to a dose *d* in grays (Gy):

(1) $$\mathrm{SF}\left(d\right)=\exp\left(-\alpha d-\beta d^{2}\right)$$

where α and β are parameters corresponding to the radiosensitivity of the irradiated tissue, which varies for each tumor subtype. In Jalalimanesh et al., the model is based on a modified LQ model representing the survival probability S of a cell irradiated with a dose *d*:

(2) $$S\left(d\right)=\exp[\gamma_{r}\left(-\alpha \;\mathrm{OMF}\;d-\beta {\left(\mathrm{OMF}\;d\right)}^{2}\right)]$$

where $\gamma_{r}$ is a factor that reflects the changes in the cell’s radiosensitivity in different phases of the cell cycle, and OMF (oxygen modifying factors) depends on the oxygenation of the cell (as hypoxic cells are less sensitive to radiation). The effect of radiation can thus be implemented by going through each cell in the simulation, applying this model, and making it survive or die depending on the predicted survival probability.

While the Jalalimanesh et al. [11] model is efficient enough to provide enough training samples for a deep learning algorithm, some important effects in the developments of cancers, such as angiogenesis, are omitted.

### 2.2. Reinforcement Learning in Radiation Therapy

Reinforcement learning (RL) is a branch of machine learning in which an agent learns to optimize its decision-making from observations of its environment and feedback on the actions that it takes [13].

In RL algorithms, the feedback that the agent receives is a scalar called the reward. The agent aims to maximize the sum of rewards over time. It is important to design how those rewards are computed very carefully, as this defines the objective of the agent. This can be notably hard in cases such as radiation therapy, where multiple objectives need to be taken into account [14].

In Tseng et al. [15], a deep reinforcement learning algorithm, deep Q network (DQN), was used to learn dose fraction adaptation in radiotherapy. Based on an initial dataset of 114 patients who received radiotherapy for lung cancer, a generative adversarial network was used to create a larger amount of training data, while a deep neural network was trained on that data to estimate transition probabilities during the treatment and thus create a radiotherapy artificial environment for the RL agent. Finally, a DQN was trained to estimate the value of each possible dose fraction. The reward function used is based on finding the best balance between a high TCP and a low NTCP.

In Jalalimanesh [11], the agent uses the tabular Q-learning algorithm, where the considered actions are three possible doses of radiation (2.5, 3, and 3.5 Gy). As observations, the number of tumor cells in the simulations is discretized into 200 stages, with a step between two stages corresponding to an increase of 25 cells. As a reward, the agent receives a scalar corresponding to the difference between tumor and healthy cells killed directly by the chosen dose, minus a factor proportional to the dose and the irradiated area.

In this article, we use DRL and study different reward functions that capture different preference functions in the treatments. Deep learning allows the use of high-dimensional features such as images as inputs, instead of making arbitrary decisions to define a state space. For our experiments, we define two different environments: (i) a simple 1D toy environment and (ii) a more realistic 2D simulation. Finally, our source code is publicly available (https://github.com/gregoire-moreau/radio_rl (accessed on 18 February 2021)), which we hope will help foster new research in this area.

## 3. Materials and Methods

### 3.1. Tumor Growth Simulation

To simulate tumor development inside healthy tissue and the effect of radiation therapy, a lattice-gas cellular automaton was implemented. This simulation is based on a 2D 50-by-50 grid where each pixel of the grid contains:

- a list of cells,
- an amount of nutrients and
- (potentially) a nutrient source.

The same cell cycle as in [10] is used: eleven hours for G1, eight hours for S, four hours for G2, and one hour for M. Healthy cells also have the ability to enter the G0 phase if the quantity of a nutrient in the pixel is too low (<q_g_G0_ for glucose or <q_o_G0_ for oxygen) or the density of the patch formed by the pixel and its neighbors is greater than 1. The simulation is initialized with one tumor cell in the center of the grid and *n*_h_cells_ randomly placed healthy cells.

The cells need two nutrients to develop: glucose and dioxygen, reactants of the aerobic respiration producing ATP that fuels the cells. Initially, a quantity *q*_g_ini_ glucose and *q*_o_ini_ dioxygen are placed for each pixel. Nutrients spread uniformly to the neighborhood at a rate of 20% per hour. Each cell’s nutrient consumption is determined at the creation for healthy cells and varied every hour for cancer cells. These consumption rates are normally distributed based on averages (μ_g_healthy_, μ_o_healthy_, μ_g_tumor_, μ_o_tumor_) and standard deviation defined as a third of the average consumption. To generate nutrients during the simulation, *n*_sources_ sources are randomly placed on pixels of the grid at the start of the simulation. Every hour, each of these sources will add *q*_g_ mg of glucose and *q*_o_ of dioxygen to the pixel on which they are placed. To simulate angiogenesis, the sources have a probability of moving to each adjacent pixel every simulated hour. They follow a random walk with a probability of moving towards the tumor’s center proportional to the number of cells forming this tumor, and a uniform probability of moving to any other adjacent pixel.

To represent the effect of radiation on cells during radiation therapy, the modified LQ model presented in Jalalimanesh et al. [11] is used (see Equation (2)). The dose is deposited using an accurate profile corresponding to the convolution product of a window function and a Gaussian. Thus, each cell is irradiated with a dose depending on its distance from the tumor’s center of mass, with cells one tumor radius away from this center receiving 95% of the total dose. The tumor’s current center of mass and radius are automatically computed after each irradiation.

In order to study first a simple environment for the RL algorithm, we built a toy environment where we collapsed the different elements of the grid to 0 dimensions. The same amounts of glucose and dioxygen distributed to the full grid initially and every hour in the 2D model are given to one pixel representing the whole environment. Initially, this pixel contains one tumor cell and healthy cells *n*_h_cells_. At each step, cells consume the nutrients to spread. During the treatment, the same dose of radiation will be applied to every cell in the simulation.

Table 1 presents the parameters used in our models.

### 3.2. Reinforcement Learning

Reinforcement learning (RL) is a branch of machine learning that aims to solve the problem of sequential decision-making. In this framework, an agent receives observations of the state of the environment in which it evolves and can choose actions to affect this environment. If we can assume that the problem is fully observable, then it can be formalized as a Markov Decision Problem (MDP), which is defined by the following elements:

- States $s_{t}\in S$, where the state space S is,
- Actions $a_{t}\in A$, where the action space A is,
- Rewards $r_{t}\in \left[0,R_{max}\right]$, where $R_{max}$ is set to 1 by scaling the rewards,
- A transition function $T\left(s,a,s^{\prime }\right):S\times A\to P\left(S\right)$ that gives the probability of the next state s’ given the current state *s* and action *a*,
- A discount factor γ∈[0,1).

At each time step, the agent observes the state of the environment and chooses an action. The effect of this action causes a transition of the environment to a new state and the agent receives a reward in the form of a scalar that indicates the quality of this transition. The agent’s goal is to maximize the sum of the rewards that it receives over time.

During its training, the agent will use trial and error to learn policies. The policy that an agent follows maps a state observed by the agent to the action that it should take (or to a distribution of actions).

The value of a state-action pair is the total amount of reward an agent can expect to accumulate over the future steps, starting from that state and taking this action according to the current policy. It can be defined mathematically with:

(3) $$q_{\pi }\left(s,a\right)≐E_{\pi }\left[\sum_{k=0}^{\infty }\;\gamma^{k}\;r_{t+k+1}|s_{t}=s,a_{t}=a\right]$$

where $E_{\pi }$ is the expected value corresponding to probabilities defined by the current policy π, the discount factor γ determines how much the immediate reward should be favored over future rewards, and k is the number of timesteps away from the current state.

A common problem encountered during the training of RL agents is the exploration/exploitation dilemma. To maximize the sum of rewards that it receives, the agent should exploit the best solutions that it has already found, but should also keep exploring the state and action spaces in order potentially to discover better strategies. A common strategy to address this problem is the ϵ-greedy algorithm, in which at each time step, the agent has a given probability ϵ of taking a random action instead of the best estimated action.

#### 3.2.1. Q-Learning

Value-based algorithms aim to solve the RL problem by learning an approximation of the optimal action-value function. This function has the form:

(4) $$Q^{*}\left(s,a\right)=\max_{\pi }q_{\pi }\left(s,a\right)$$

where $q_{\pi }\left(s,a\right)$ is the value of a state-action pair (*s*,*a*) under the current policy π and $Q^{*}\left(s,a\right)$ is the value of this pair under an optimal policy. Therefore, $Q^{*}\left(s,a\right)$ is the maximum discounted sum of rewards that could be expected under an optimal policy, assuming that the agent has just observed state *s* and taken action *a*. If the agent has access to this function, it can trivially reach optimal decision-making simply by choosing action $a^{*}={\mathrm{argmax}}_{a}Q^{*}\left(s,a\right)$ in each state s.

Tabular Q-learning uses a table containing an entry for each possible state-action pair to approximate $Q^{*}$. This means that the algorithm requires the state-action space to be discrete and small to produce accurate estimations. At each time step, the agent observes the current state *s* of the environment, chooses an action *a* to take depending on its learning strategy, and receives a reward *r* once the action has been applied. This process repeats at the next time steps when observing the new state $s^{\prime }$ that was reached. It will then update recursively the entry in the table corresponding to the initial state and the action that was chosen with:

(5) $$Q\left[s,a\right]=\left(1-\alpha \right)Q\left[s,a\right]+\alpha (r+\gamma \max_{a^{\prime }}Q\left[s^{\prime },a^{\prime }\right])$$

where α is the step-size parameter that can be decreased during the training once the estimations become more accurate.

In deep Q-learning, a neural network is used to approximate $Q^{*}$. It receives observations of the environment’s state as inputs, and estimates the value of each possible action as an output. At each step in the training algorithm, the values given by the neural network $Q(s,a|\theta^{Q})$ for state *s*, action *a*, and parameterized $\theta^{Q}$ are updated towards a target value given by the formula:

(6) $$y_{t}=r_{t}+\gamma \max_{a^{\prime }\in A}Q\left(s^{\prime },a^{\prime }|\theta^{Q}\right)$$

In DQNs [16], two additions help with the stability of the training. First, a large buffer of past experiences is kept so that the agent can train on batches sampled randomly from this buffer instead of using only the most recent examples. This trick, called experience replay, ensures that training samples are more independent from each other. Second, the targets used to estimate the loss are computed using a second “target” network, to increase the stability of these targets. This network is obtained by cloning the main network once every *C* training steps, where *C* is usually at least of the order of $10^{4}$.

#### 3.2.2. Deep Deterministic Policy Gradient

Policy-based algorithms aim instead to estimate the agent’s policy directly, and to improve it over time. The deep deterministic policy gradient (DDPG) algorithm trains two different networks [17] simultaneously. The actor network approximates the function μ(s), which defines the agent’s policy; it receives observations of the environment’s state as inputs, and outputs the action that the agent should take. The critic network approximates the function Q(s,a), which is used to evaluate the actions chosen by the agent. It receives both an observation of the environment’s state, and the action that the agent chose in this state, and outputs an estimation of the expected return when taking this action in that state, while following μ(s) afterwards. Compared with DQN, DDPG has the possibility to work with a continuous action space.

The critic is trained to be increasingly accurate in its estimations of action-values. It uses a training target with the form:

(7) $$y_{i}=r_{i}+\gamma Q\left(s^{\prime },\mu \left(s^{\prime }|\theta^{\mu }\right)|\theta^{Q}\right)$$

The actor is trained with a policy gradient. This gradient uses the critic and has the form:

(8) $$\nabla_{\theta^{\mu }}J=\frac{1}{N}\sum_{i}\nabla_{a}Q\left(s,a|\theta^{Q}\right)\left|s=s_{i},a=\mu \left(s_{i}\right)\nabla_{\theta^{\mu }}\mu \left(s|\theta^{\mu }\right)\right|s_{i}$$

The policy will move towards actions that lead to higher values. As the actions are directly predicted by the actor, the action space is continuous and can be multi-dimensional. This algorithm is therefore more flexible than DQN.

Experience replay and target networks are also used to train the critic in DDPG to improve stability.

### 3.3. Integration with the Simulation

In order to use RL algorithms with the previously described models, the different elements of an MDP have to be defined in terms of these models.

**Figure 2:**
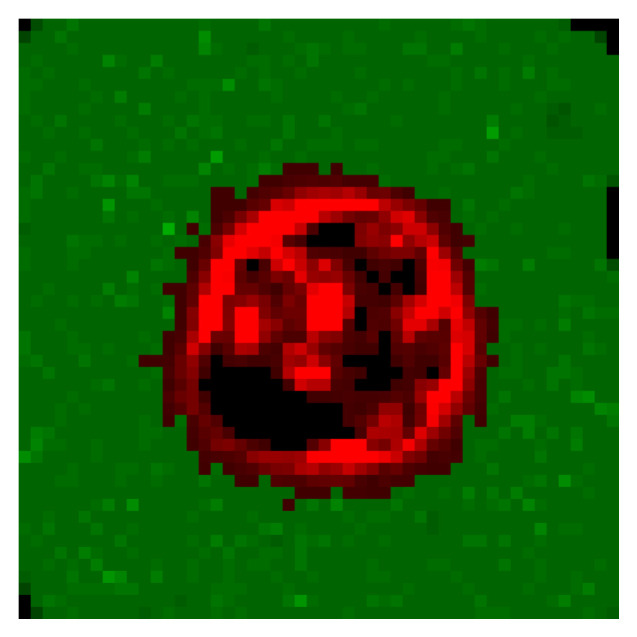
Observation of the 2D model where green pixels are healthy cells, red pixels are tumor cells, and black pixels are empty volumes. The intensities are proportional to the density of cells.

Observations are used by the agent as the current state of the simulation. In the collapsed scalar model, this state is made out of a combination of the number of cancer cells and healthy cells after they have been discretized. The DRL algorithms used with the 2D model allow for richer observations. Here, they are 50-by-50 images, where each pixel’s value is either the number of cancer cells on this pixel on the grid, if there are any, or the number of healthy cells on this pixel. Figure 2 shows such an observation.

The actions chosen by the agent define the EBRT treatment schedule applied to the simulation. For all algorithms, the agent will choose the dose with which to irradiate the tumor at each time step. This dose will be chosen in the interval [1–5] Gy. This domain is discrete for Q-learning algorithms, with steps of 1 Gy for tabular Q-learning and 0.5 Gy for DQN. It is continuous for DDPG. Furthermore, agents using the DDPG algorithm will also choose the time between the current fraction and the next fraction, in a domain of 12–72 h.

After the action chosen by the agent has been applied to the environment, it will receive a reward indicating the quality of this action. The agents have been trained to optimize two different reward functions: “killed” (K) and “killed and dose” (KD). On regular (non-terminal) time steps, agents will receive rewards of the form:

- Killed:
  (9) $$\frac{\#\;\mathrm{cancer}\;\mathrm{cells}\;\mathrm{killed}\;-\;x\;\times \;\#\;\mathrm{healthy}\;\mathrm{cells}\;\mathrm{killed}}{y}$$
- Killed and dose:
  (10) $$\frac{\#\;\mathrm{cancer}\;\mathrm{cells}\;\mathrm{killed}\;-\;x\;\times \;\#\;\mathrm{healthy}\;\mathrm{cells}\;\mathrm{killed}}{y}-\frac{\mathrm{radiation}\;\mathrm{dose}}{z}$$

In the 2D model, the values of the constants *x*, *y*, and *z* are 5, 100,000, and 200, respectively.

On terminal steps, agents will receive rewards of -1 if the treatment was unsuccessful, or of the form:

- Killed:
  (11) $$0.5-\frac{\#\;\mathrm{initial}\;\mathrm{healthy}\;\mathrm{cells}\;-\;\#\;\mathrm{final}\;\mathrm{healthy}\;\mathrm{cells}}{k}$$
- Killed and dose:
  (12) $$0.5-\frac{\#\;\mathrm{initial}\;\mathrm{healthy}\;\mathrm{cells}\;-\;\#\;\mathrm{final}\;\mathrm{healthy}\;\mathrm{cells}}{k}-\frac{\mathrm{radiation}\;\mathrm{dose}}{z}$$

In the 2D model, the values of the constants *k* and *z* are 3000 and 200, respectively. The first reward function “Killed” was designed to correspond to the objective of maximising damage to cancer cells while minimising the damage to healthy cells, while the second reward function, “Killed and dose”, adds a parameter, leading the agent to find treatments with smaller total doses, as long term effects related to this total dose are not modelled in the simulation. At positive terminal steps, rewards correspond to the state of healthy tissue after the tumor has been eliminated. Parameters x, y, z and k were chosen experimentally as they lead to better stability of the algorithms.

A simulated treatment is considered finished in three possible situations. First, the treatment will have succeeded if the tumor has been destroyed and no cancer cells are left in the model. Second, the treatment will have failed if the tumor has completely invaded healthy tissue, and there are fewer than 10 healthy cells in the model. Third, the treatment will have timed out if the two previous conditions are not reached after 1200 h of treatment.

### 3.4. Evaluation of Performances

For each combination of environment, algorithm, and reward function, 1000 test treatments are generated. The performances of each treatment are assessed using several indicators:

- TCP
- Total radiation dose (D_mean_)
- Number of fractions (N_fract_)
- Treatment time (T_mean_)

NTCP criteria are not included as a performance indicator because of the absence of organs at risk in the model, making the estimation of side effects impossible. To put these performance indicators into context, they will be compared with a baseline corresponding to a dose of 2 Gy every 24 h, as in a conventional RT treatment. The sample mean of each performance indicator is given along with its standard error when relevant. Furthermore, to illustrate the policies found by agents to optimize their reward functions, a graph will give the mean and standard deviations of the radiation dose prescribed at each step of the treatment.

## 4. Results

### 4.1. Collapsed Toy Environment

Table 2 presents the performances of the agents trained on the collapsed toy environment using the tabular Q-learning algorithm.

We observe that for both reward functions (K and KD), a better TCP can be obtained using fewer fractions and lower radiation doses. While the baseline treatment needed 39 fractions and an average dose of 77.9 Gy to reach a TCP of 97%, agents trained to optimize the K (and the KD) reward function need 32.2 (and 27.7) fractions and an average dose of 66.7 Gy (and 61.2 Gy) to reach a TCP of 100% (100%).

**Figure 3:**
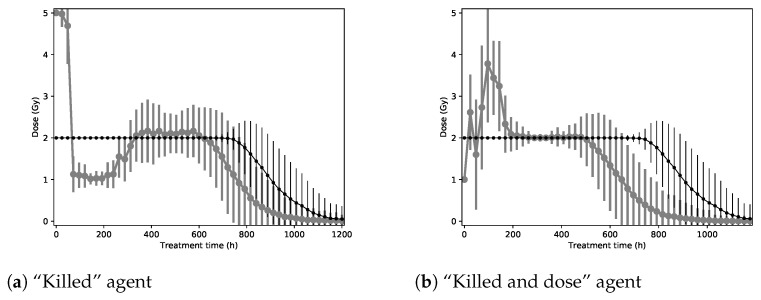
Shape of treatments found by the agent for the collapsed toy environment (in gray) compared with the baseline treatment (in black). Each point represents a dose delivered to the patients.

Figure 3a shows the shape of the treatments used on the toy environment by the agent that optimized the “Killed” reward function. We can see that three high radiation doses are delivered at the beginning to eliminate a large portion of the cancer cells. Then, lower doses are used to destroy the remaining cancer cells, while allowing healthy cells to recover. Figure 3b shows the shape of the treatments used on the toy environment by the agent that optimized the “Killed and dose” reward function. The agent always starts with a dose of 1 Gy. In the few following steps, the observed doses have a very high standard deviation, indicating a high sensitivity to the situation. The agent will then tend to destroy the remaining cancer cells in the model using doses of around 2 Gy.

### 4.2. 2D Environment

Table 3 presents the performances of the agents trained on the 2D model using the DQN and DDPG algorithms.

Here again, we observe that for both combinations, a better TCP (100% versus 99%) can be obtained using a lower number of fractions and a lower dose. The shorter treatment is the one obtained using DQN and the “Killed and dose” reward function (8.2 fractions, 32.1 Gy). It uses half the dose sent by the baseline and one-quarter as many fractions are required.

**Figure 4:**
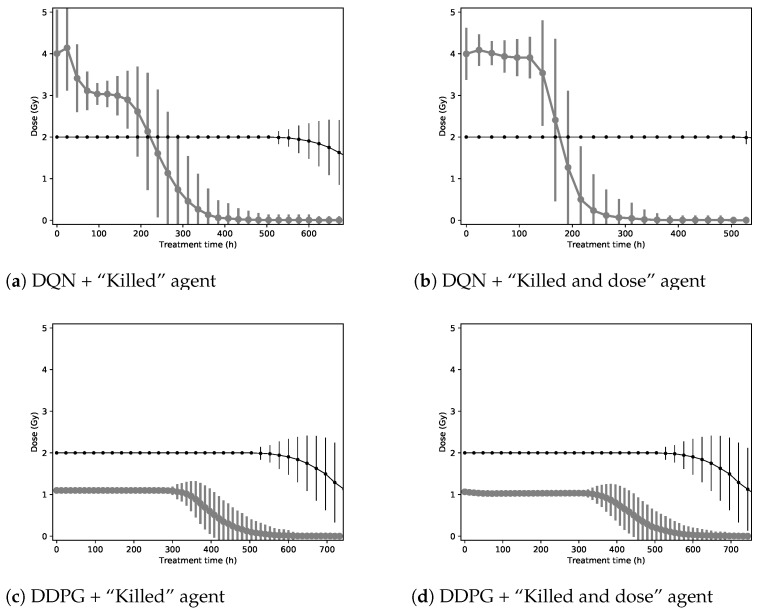
Shape of treatments found by the agents for the 2D environment (in gray) using the DQN algorithm (**top**) and the DDPG algorithm (**bottom**). The baseline is shown (in black) for comparison. Each point represents a dose delivered to the patients.

Figure 4 shows the shape of the treatments used on the 2D environment by the agent that optimized the “Killed” and “Killed and dose” reward functions, using the DQN and the DDPG algorithms.

Concerning the DQN algorithm with the “Killed” function (see Figure 4a), the agent sends a few high doses at the start of the treatment, then destroys the remaining cancer cells with daily doses of around 3 Gy. For the “Killed and dose” function (see Figure 4b), the agent chooses to send high doses throughout the treatments.

Concerning the DDPG algorithm with the “Killed” function (see Figure 4c), the agent chooses to deliver a dose slightly higher than 1 Gy every 12 h for the entire treatment. For the “Killed and dose” function (see Figure 4d), like the other DDPG agent, this agent chooses to deliver a dose slightly higher than 1 Gy every 12 h throughout the treatment.

**Figure 5:**
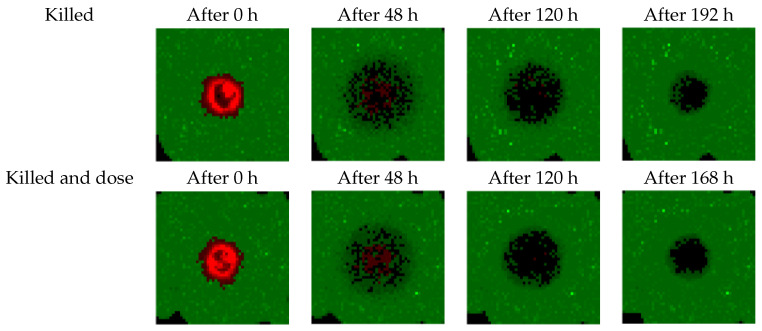
Effects of the treatments prescribed by the DQN agents on the 2D simulation using the two studied reward functions (K and KD). Green pixels are healthy cells, red pixels are tumor cells, and black pixels are empty volumes.

Figure 5 shows the effects of treatments suggested by the agents controlled by the DQN algorithm on the simulation (see Figure 4a,b). The first few doses of the treatments impact a large share of the tumor and surrounding tissues. After 120 h, only a few cancer cells remain and healthy tissue is starting to recover from the initial radiation damage. Finally, at the end of the treatments, there are no more cancer cells on the grid, while healthy tissue has completely recovered from the treatment.

**Figure 6:**
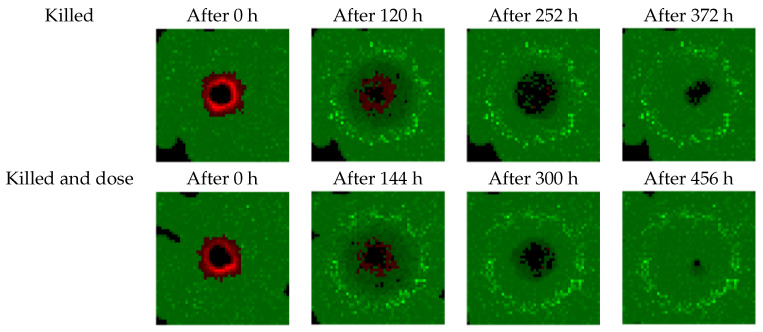
Effects of the treatments prescribed by the DDPG agents on the 2D simulation using the two studied reward functions (K and KD). Green pixels are healthy cells, red pixels are tumor cells, and black pixels are empty volumes.

Figure 6 shows the effects of treatments suggested by agents using the DDPG algorithm on the simulation (see Figure 4c,d). The low but frequent doses initially stop the tumor’s growth and cause it some damage, while mostly sparing healthy tissue. Two-thirds of the way into the treatments, cancer cells have mostly been destroyed and only healthy cells close to the initial borders of the tumor have been impacted by the radiation. At the end of the treatments, healthy tissue has mostly recovered the space invaded by the tumor before these treatments started.

## 5. Discussion

Our experiments have shown that using reinforcement learning is a promising option for the EBRT dose fractioning automation. As suggested by Jalalimanesh et al. [11], a model simulation is a good alternative to model the tumor growth necessary for RL training. Because this kind of model is flexible, we chose to complexify it compared with the previous articles by studying the impact of both glucose and oxygen in the same model, and by modeling effects such as angiogenesis. Furthermore, we studied the performances of several RL algorithms (tabular Q-learning, DDPG and DQN). In particular, the different deep RL approaches allowed us to consider different action spaces, namely, (i) a fixed set of actions (DQN) and (ii) a continuous action space (DDPG).

It is necessary to specify that finding the optimal policy is difficult because of the model’s complexity, in particular, its high-dimensional state space, stochasticity, and nonlinear dynamics. The impact of several random parameters is also quite important (e.g., initial positions of healthy cells, nutrient consumption of cells, positions of daughter cells, probability of linear survival for large numbers, but not when only a few cancer cells remain, repair time in case of survival, and sources, positions and displacement). This point explains the significant standard deviation in our performance indicators. However, even despite this, our RL algorithms could converge.

Our deep RL agents are able to obtain better treatment schedules than a simple baseline with (i) improved TCP, (ii) shorter treatments, and (iii) lower overall radiations. The improvement is particularly important for the 2D environment. In our simulations, we have observed that starting with relatively high doses and decreasing the radiation doses through the treatment improves the cumulative rewards. We have also observed that hyper-fractionation might lead to better treatments. Further studies with an even more realistic in silico model or clinical trials would be of interest to validate these observations.

The two different reward functions produce slightly different results when measured with performance indicators. Indeed, the added dose factor in the “Killed and dose” reward function creates agents that suggest on average shorter treatments with a lower radiation dose and number of fractions than the “Killed” reward function. This effect is observed for the tabular Q-learning and DQN algorithms.

Agents trained with the DDPG algorithm reached the same treatment patterns while optimizing both reward functions. These treatments (sending close to 1 Gy every 12 h) always use actions very close to the minimum for each dimension of the action space. Different treatments might thus be found if the action space is extended below the current minimum.

Our study has some limitations. The cellular model is not realistic enough and needs several improvements. For instance, as shown in Figure 5 and Figure 6, the model overestimates healthy tissue recovery. Another example is the lack of consideration of the acute tolerance (nausea, vomiting, dysphagia) according to the dose per fraction. The model neglects the chemosensitization and the revascularization as well. Moreover, the model instantly deletes cells that are considered to have been killed by radiation. We also used an “almost” perfect dose profile. The radiation beam is able to target tumor cells accurately while avoiding healthy cells.

Future work in several directions is needed to bring this research work closer to a useful software for the doctors: (i) The in silico model developed for this paper is rather generic and could benefit from an improved cell model that would take the specificities of the different organs and different tumor types into account. (ii) Medical doctors should help develop improved heuristics in terms of the rewards optimized through reinforcement learning, while data from clinical trials should ultimately allow for optimizing directly for the improvement in patient life expectancy. (iii) A transfer learning approach from CT scans to the in silico model should be developed in order to have personalized treatment planning in real time.

## 6. Conclusions

In this paper, we studied how the use of deep reinforcement learning can be used to simulate dose fractionation based on a tumor growth model. A dose planning algorithm applied to a mathematical model of healthy tissue and tumor growth took damage to both healthy tissue and the tumor into account in order to optimize the fractions. Two different deep RL algorithms were trained, each with two different preference functions. Compared with a baseline of current practice, the treatments found by the DRL agents outperformed in the selected study criteria.

The purpose of this work is to automate parts of treatment planning which are currently being designed by human experts. Thanks to radiomics features extracted from CT scans and a biological in silico model of radiation effects, an RL approach could pave the way to adaptive treatments that are able to take the specifics of each patient’s case (tumour shrinkage, deformations, etc.) into account.

## Figures and Tables

**Table 1:** Growth model parameters.

Parameter	Notation	Value
Glucose quiescence level	$q_{g\_G0}$	$1.728\times 10^{-7}$ mg/cell
Oxygen quiescence level	$q_{o\_G0}$	$10.37\times 10^{-8}$ mL/cell
Starting healthy cells	*n*_h_cells_	1000
Starting nutrient sources	*n*_sources_	100
Starting glucose	*q*_g_ini_	$1\times 10^{-6}$ mg
Starting oxygen	*q*_o_ini_	$1\times 10^{-6}$ mL
Glucose average consumption	μ_g_healthy_	$3.6\times 10^{-9}$ mg/cell/hour
	μ_g_tumor_	$5.4\times 10^{-9}$ mg/cell/hour
Oxygen average consumption	μ_o_healthy_	$2.16\times 10^{-9}$ mL/cell/hour
	μ_o_tumor_	$2.16\times 10^{-9}$ mL/cell/hour
Glucose inputs per step	*q*_g_	$1.3\times 10^{-6}$ mg/source/hour
Oxygen inputs per step	*q*_o_	$4.86\times 10^{-7}$ mL/source/hour

**Table 2:** Results of reinforcement learning trained on the collapsed toy environment using the tabular Q-learning algorithm (mean ± standard error).

Rewards *	TCP (%)	D_mean_ (Gy)	N_fract_	T_mean_ (h)
Baseline	97	77.9 ± 0.3	39 ± 0.2	934.8 ± 3.8
K	100	66.7 ± 0.3	32.2 ± 0.1	771.9 ± 3.3
KD	100	61.2 ± 0.3	27.7 ± 0.1	664.4 ± 3.6

* K for killed, KD for killed and dose.

**Table 3:** Results of reinforcement learning trained on the 2D environment using two different RL algorithms and two different reward functions (mean ± std).

	Rewards *	TCP (%)	D_mean_ (Gy)	N_fract_	T_mean_ (h)
Baseline	/	99	65.6 ± 0.3	32.8 ± 0.2	787.7 ± 4
DQN	K	100	35.6 ± 0.2	10.9 ± 0.1	262.2 ± 1.6
DQN	KD	100	32.1 ± 0.2	8.2 ± 0.1	195.8 ± 1.4
DDPG	K	100	38.5 ± 0.2	35.1 ± 0.2	420.9 ± 2.1
DDPG	KD	100	39.4 ± 0.2	38.1 ± 0.2	457.1 ± 2.2

* K for killed, KD for killed and dose.
